# Quantum Mechanisms of Electron and Positron Acceleration through Nonlinear Compton Scatterings and Nonlinear Breit-Wheeler Processes in Coherent Photon Dominated Regime

**DOI:** 10.1038/s41598-019-55472-5

**Published:** 2019-12-11

**Authors:** Bo Zhang, Zhimeng Zhang, Zhi-gang Deng, Jian Teng, Shu-kai He, Wei Hong, Weimin Zhou, Yuqiu Gu

**Affiliations:** 1 Department of High Energy Density Physics, Research Center of Laser Fusion, 621900 Mianyang, Sichuan China; 2 Laboratory of Science and Technology on Plasma Physics, Research Center of Laser Fusion, 621900 Mianyang, Sichuan China

**Keywords:** Laser-produced plasmas, Plasma-based accelerators

## Abstract

Electric force is presently the only means in laboratory to accelerate charged particles to high energies, corresponding acceleration processes are classical and continuous. Here we report on how to accelerate electrons and positrons to high energies using ultra intense lasers (UIL) through two quantum processes, nonlinear Compton scattering and nonlinear Breit-Wheeler process. In the coherent photon dominated regime of these two processes, the former can effectively boost electrons/positrons and the latter can produce high energy electrons and positrons with low energy *γ* photons. The energy needed for such quantum acceleration (QA) is transferred from large numbers of coherent laser photons through the two quantum processes. QA also collimate the generated high energy electrons and positrons along the laser axis and the effective acceleration distance is of microscopic dimensions. Proof of principle QA experiment can be performed on 100 petawatt (PW) scale lasers which are in building or planning.

## Introduction

Since invented in the early 1930s, accelerators used electric fields to boost charged particles. For example, the accelerating fields in conventional accelerators were waves generated by radio frequency cavities; Plasma based accelerators employed plasma waves (wakefields) excited by laser pulses^1–3^ or charged particle bunches^4–6^ to propel electrons or positrons.

In contrast to these classical and continuous acceleration methods based on electric force, quantum processes, e.g., binary scatterings, appear hardly suitable for particle acceleration. To boost a particle to a high energy scale, binary scatterings usually need a driver particle that has already reached that energy scale, i.e., unworthy. Furthermore, their cross-sections are usually too small, i.e., low probabilities.

However, if the scattering is driven by a large number of coherent particles, particle acceleration through quantum processes is possible for both problems can be solved. The energy of a single driver particle can be much lower than the target energy scale. The probabilities of such scatterings can be much higher for they usually grow nonlinearly with the density of coherent driver particles and other parameters.

UILs are the most powerful coherent particle sources in laboratory today. Present record of laser intensity *I* is 2 × 10^22^ W/cm^2^ ^7^ and it is anticipated to reach 10^23–25^ W/cm^2^ on 100 PW scale laser facilities such as ELI^8^, XCELS^9^ and SEL^10^ which are in planning or building. EW scale laser which in theory can reach 10^26^ W/cm^2^ is also the trend of future developments.

As will be presented in this paper, there are two methods to accelerate electrons and positrons to high energies through quantum processes with UILs, Quantum Acceleration through Nonlinear Compton Scattering (QANCS) and Quantum Acceleration through Nonlinear Breit-Wheeler process (QANBW). The former boosts electrons and positrons and the latter creates high energy electron-positron pairs with low energy *γ* photons. They both transfer the energy and momentum of coherent laser photons to high energy electrons and positrons discretely and collimate them along the laser axis.

## Basics of Quantum Acceleration

QANCS is the coherent photon dominated case of nonlinear Compton scattering (NCS)

$${e}^{-}(p)+n{\omega }_{L}(k)\to \gamma (k^{\prime} )+{e}^{-}(p^{\prime} ),$$

where *e* is the electron, *ω*_*L*_ the laser photon, *γ* the emitted photon and *p*, *k*, *k*′ and *p*′ their 4-momenta (energy-momentum). The differential probability of NCS *dW*/*dudt* is given in Eq. (8) in the Method part.

In UIL fields, NCS has very short coherence interval therefore should be considered as a instantaneous and local process. On existing laser facilities, the total energy of laser photons involved in a single NCS is usually much lower than that of the electron therefore is negligible. As a result, present researches usually assume ${k}_{0}^{\prime} +{p}_{0}^{\prime} ={p}_{0}+n{k}_{0}\approx {p}_{0}$ in simulations^11–13^, where *p*_0_ is the electron energy before scattering, *p*′_0_ is that after scattering, etc. However, when the energy of involved laser photons exceed that of the electron (usually at $I\gtrsim {10}^{24}$ W/cm^2^), the coherent photons would dominate the scattering^13^. Boosting electrons or positrons by transferring part of the involved laser photon energy through scattering, i.e., ${p}_{0}^{^{\prime} } > {p}_{0}$, then becomes possible.

**Figure 1:**
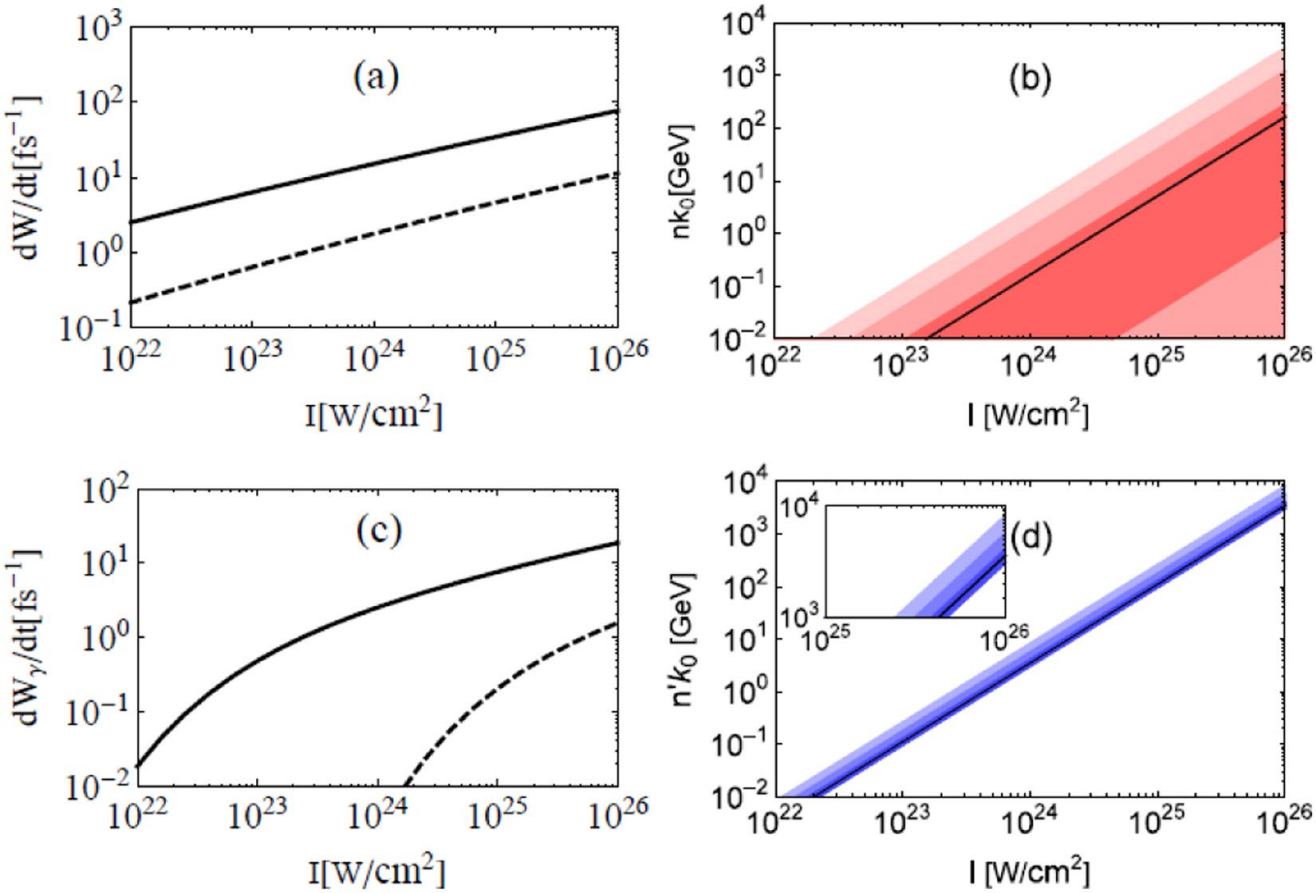
Scattering rate of an electron (**a**) and annihilation rate of a *γ* photon (**c**) at the peak of an UIL and energy of laser photons involved in a single nonlinear scattering (**b**) and annihilation (**d**). *p*_0_ and *k*′′_0_ = 1 GeV, the wavelength *λ*_0_ = 1 *μ*m, the angle to the laser axis is 30° (dashed) and 180° (solid) in (**a**) and (**c**). In (**b**) and (**d**), *χ* and *χ*_*γ*_ are fixed at 1, the solid lines are the averages ($\bar{n}{k}_{0}$ and $\bar{n}^{\prime} {k}_{0}$) and the 3 surrounding regions cover the 1*σ* (68.3%), 2*σ* (95.5%), and 3*σ* (99.7%) ranges of the distribution.

The number of laser photons involved in a single NCS^13–15^,

1 $$n\approx \frac{u}{\chi }{a}_{0}^{3},$$

depends on laser intensity, it scales as the cube of normalized laser amplitude *a*_0_ ≡ *eE*_*L*_/*mω*_*L*_, where *e* and *m* are charge and mass of electron, *E*_*L*_ the laser electric field and *ω*_*L*_ the frequency. Scattering parameters $u=kk^{\prime} /kp$ and $\chi =e\sqrt{-{({F}^{\mu \nu }{p}_{\nu })}^{2}}/{m}^{3}\approx {a}_{0}{p}_{0}{k}_{0}(1-\,\cos \,\theta )/{m}^{2}$ are of the same scale^13,14^, hence the energy of involved laser photo*n*s *nk*_0_ grows superlinearly with laser intensity. As shown inFig. 1(b), *nk*_0_ can reach 10−100 GeV when laser intensity *I*[10^18^ W/cm^2^] = 1.37*a*_0_^2^*λ*^*−*2^[*μm*] of a *λ*_0_ = 1 *μ*m laser reaches 10^25^ W/cm^2^. At 10^26^ W/cm^2^, it can reach 100 GeV to TeV. In Fig. 1(b), the average number of involved laser photons

2 $$\bar{n}=\frac{{\int }_{0}^{\infty }\,dundW/dudt}{{\int }_{0}^{\infty }\,dudW/dudt},$$

and the 1*σ* to 3*σ* ranges are the center parts of the distribution, e.g., the 1*σ* range (*n*_1*σ*−_, *n*_1*σ* +_) is defined as

3 $$\frac{{\int }_{0}^{{n}_{1\sigma \pm }}\,dn\frac{dW}{dudt}\frac{du}{dn}}{\int \,du\frac{dW}{dudt}}=\frac{1\pm 68.3 \% }{2}\mathrm{}.$$

Similarly, QANBW is the coherent photon dominated case of the symmetric process called nonlinear Breit-Wheeler process (NBW)

$$\gamma (k^{\prime\prime} )+n^{\prime} {\omega }_{L}(k)\to {e}^{-}(p^{\prime\prime} )+{e}^{+}(p^{\prime} ),$$

which annihilates a *γ* photon and creates an electron-positron pair. When the energy of involved coherent photons exceed that of the *γ* photon and dominate this process, it is possible to generate high energy electrons and positrons by annihilate low energy *γ* photons. The needed energy is also transferred from the coherent laser photons. The differential probability of NBW *dW*′/*dudt* is given in Eq. (13) in the Method part.

To effectively accelerate electrons or electron-positron pairs through QANCS/QANBW, both the scattering/annihilation rate and the energy of involved coherent photons are important. The extreme density and coherence solve both. Figure 1(a) shows the scattering rate *dW*/*dt* of a 1 GeV electron, which can be generated with state-of-art laser wakefield accelerators^2^, at the peak of an UIL. Since *dW*/*dt* increases with the angle *θ* to the laser, *θ* = 180° is the most active case and *θ* = 30° is a very inactive case. It shows that a GeV scale electron usually scatters several to dozens of times when propagating through the focus of an UIL which is of *μ*m scale (1 fs ·*c* = 0.3 *μ*m).

The annihilation rate of a 1 GeV photon and involved laser photon energy are shown in Fig. 1(c,d). The former is lower than that of electron in Fig. 1(a), and the latter is of the same scale as that of electron shown in Fig. 1(b). The number of laser photons involved in a single NBW

4 $$n^{\prime} \approx \frac{1}{\delta (1-\delta ){\chi }_{\gamma }}{a}_{0}^{3}$$

has a much narrower distribution and a clear lower bound at ${a}_{0}^{3}/{\chi }_{\gamma }$^13,14^, where ${\chi }_{\gamma }=e\sqrt{-{({F}^{\mu \nu }{k}_{\nu }^{^{\prime} })}^{2}}/{m}^{3}$ is a key parameter for this annihilation. The average and range of *n*′ in Fig. 1(d) have similar definitions to Eqs. (2) and (3).

**Figure 2:**
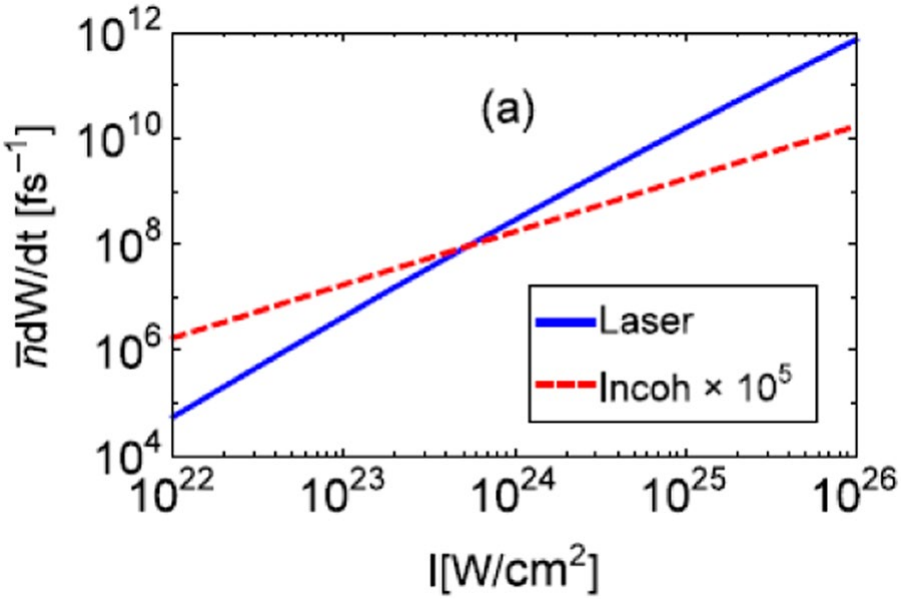
Expectation of laser photon scattering rate of an electron in an UIL (blue solid) or in an incoherent and otherwise similar photon beam (red dashed). *λ*_0_ = 1 *μ*m, electron energy is 1 GeV and the angle is *θ* = 30°.

Coherence is crucial for such high energy. Figure 2 shows the average photon scattering rate $\bar{n}dW/dt$of an electron. When the intensity reaches 10^24^ W/cm^2^ or even higher, the photon scattering rate is at least 5−6 magnitudes higher than that with an incoherent and otherwise similar photon beam. The cross section of Compton scattering can be found in ref. ^16^ and the Method part.

**Figure 3:**
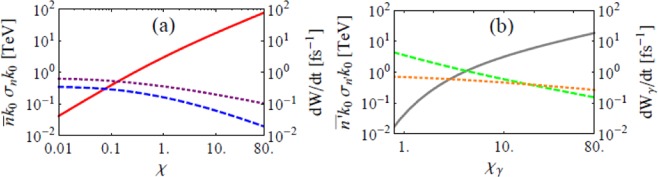
The rate of QANCS/QANBW and involved laser photon energies at the peak of an UIL. Rates of QANCS (**a**) and QANBW (**b**) are red and gray solid lines. Expectations of involved laser photon energies ($\bar{n}{k}_{0}$ and $\bar{n}^{\prime} {k}_{0}$, blue and green, dashed) and their divergences (*σ*_*n*_*k*_0_ and *σ*_*n*′_*k*_0_, r.m.s., purple and orange, dotted) were also given. Parameters are *λ* = 1 *μ*m, *I* = 10^26^ W/cm^2^ and *p*_0_ and ${k^{\prime\prime} }_{0}\mathrm{=1}$ GeV.

The scattering rate and involved laser photon energy are both important for QANCS. However, as Fig. 3(a) shows, the former grows monotonically with *χ* while the latter decreases with it. Since the energy scale of QANCS is determined by the latter while a moderate scattering rate that can ensure most electrons get QAed is enough, $0.1 < \chi /{p}_{0}$1 would be the best range for QANCS. The rate of QANBW and involved laser photon energy have similar trend as Fig. 3(b) shows, for similar reasons, the best QANBW range would be $1\lesssim {\chi }_{\gamma }\lesssim 10$. The divergence in Fig. 3(a) is

5 $${\sigma }_{n}=\frac{{\int }_{0}^{\infty }\,du{n}^{2}\frac{dW}{dudt}}{{\int }_{0}^{\infty }\,du\frac{dW}{dudt}}-{\bar{n}}^{2},$$

and *σ*_*n*′_ has similar definition.

## Quantum Acceleration

**Figure 4:**
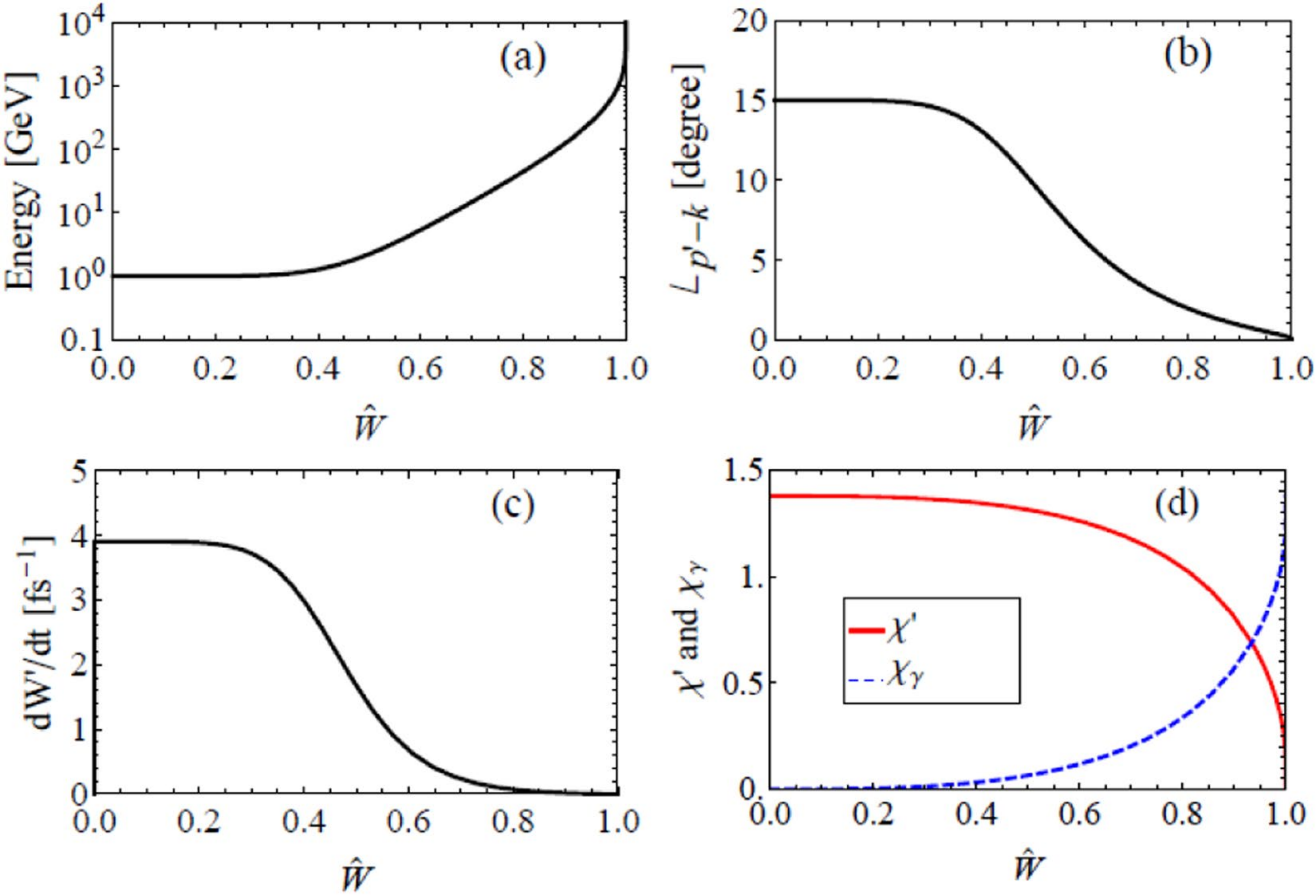
Basic features of a single QANCS. Energy distribution of electrons QAed through NCS (**a**), angle to the laser axis ∠_*p*′−*k*_ (**b**), re-QANCS rate (**c**) and *χ*′ − *χ*_*γ*_ (**d**). Parameters are *E* = 1 GeV, *θ* = 15°, *ϕ* = 90° and *I* = 10^26^ W/cm^2^.

Since the energy of coherent laser photons involved in QANCS has a wide distribution, a single QANCS does not always transfer a very high energy to the electron. Figure 4(a) shows the energy of an electron *p*′_0_(*u*_0_) QAed at the peak of an *I* = 10^26^ W/cm^2^ laser through NCS as a function of the normalized accumulated scattering rate

6 $$\hat{W}({u}_{0})=\frac{{\int }_{0}^{{u}_{0}}\,du\frac{dW}{dudt}}{{\int }_{0}^{\infty }\,du\frac{dW}{dudt}}\in \mathrm{[0},1].$$

The energy transferred to the electron grows with $\hat{W}$, high gain QANCSs (>100 GeV in this case) have a chance of ≈15%. In the rest cases, the energy gain is lower (<100 GeV).

High energy transfer is accompanied by high momentum transfer. Electrons boosted to very high energy also obtain a large momentum through the scattering therefore are collimated along the laser axis simultaneously. Figure 4(b) shows such QANCS induced collimation, the angle of QAed electron to the laser axis decreases with $\hat{W}$ and hence the electron energy. High gain QANCSs compress involved electrons within 1.5° to the laser. Formulae for QANCS induced collimation are given in the Supplemental Information.

Although a single QANCS only kicks a fraction of the electrons to very high energy, e.g., the 15% above 100 GeV, successive QANCSs strongly prefer the rest. Figure 4(c) gives the re-QANCS rate

7 $$\frac{dW^{\prime} }{dt}={\int }_{0}^{\infty }\,du^{\prime} {F}_{\chi ^{\prime} ,{p}_{0}^{^{\prime} }}(u^{\prime} )$$

of an electron QAed through NCS, where $\chi ^{\prime} =e\sqrt{-{({F}^{\mu \nu }{p}_{\nu }^{^{\prime} })}^{2}}/{m}^{3}$ and *F*_*χp*0_ is given in Eq. 8. It shows that, after a low gain QANCS, the electron is highly possible to experience more QANCSs while a high gain QANCS strongly suppresses successive scatterings. This suppression is due to enhanced electron energy and the decreased *χ* shown in Fig. 4(d). Such selective mechanism keeps successfully QAed electrons from losing energy through further scatterings while keep on boosting the rest.

After an electron is kicked by QANCS to very high energy at the focus, the electron co-propagates with the laser since it is also well collimated along the laser axis. In the co-propagation process, the electron is mainly influenced by radiation and Lorentz force. The main mechanism of the former is NCS and is highly suppressed then, and the electric and magnetic terms of the latter almost cancel with each other. As a consequence, the electron is kept almost intact in the co-propagation process. With a mirror that deflects the laser at macroscopic distance, the beam can be safely separated from the laser. For detailed analysis of the co-propagation and separation, see the Supplemental Info.

**Figure 5:**
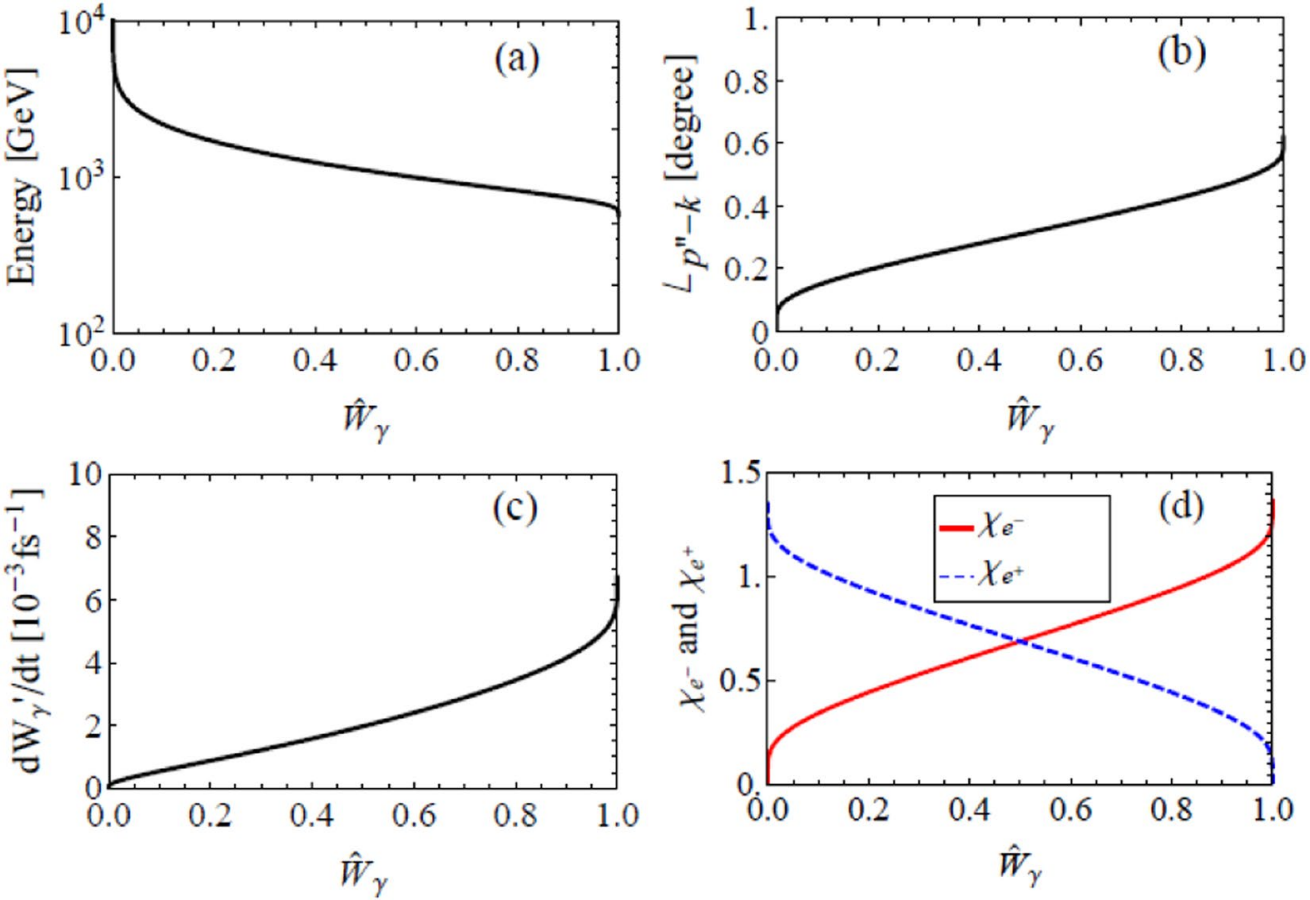
Basic features of a single QANBW. Energy of the electron QAed through NBW (**a**), angle to the laser ∠_*p*′′−*k*_ (**b**), re-scattering rate (**c**) and *χ* of created charges (**d**). Parameters are *E*_*γ*_ = 1 GeV, *θ* = 15°, *ϕ* = 90° and *I* = 10^26^ W/cm^2^, and the positron results are symmetric with respect to ${\hat{W}}_{y}$ = 0.5.

Besides kicking the electrons, QANCS also emit *γ* photons which can trigger QANBWs in the UIL. Figure 5 shows the QANBW of a 1 GeV *γ* photon, parameters are similar to the QANCS shown in Fig. 4.

A very important difference from QANCS is that, the energy of laser photons *n*′*k*_0_ involved in a single QANBW and therefore the energies of created *e*^−^ and *e*^+^ have a lower bound as shown in Fig. 5(a), where ${\hat{W}}_{\gamma }\in \mathrm{[0,}\,\mathrm{1]}$ is the normalized accumulated probability of NBW which has similar definition to Eq. (6). Besides high energy electron-positron creation, the lower bound of *n*′ has two additional effects. First, the momentum of involved photons is always overwhelming, created electron and positron are hence tightly collimated along the laser axis (<0.6°) as shown in Fig. 5(b). Second, it strongly suppresses the scattering between the created charges and the laser as shown in Fig. 5(c). Note that the energy, angle and re-scattering rate of positron are symmetric to that of the electron in Fig. 5(a–c) with respect to ${\hat{W}}_{\gamma }=0.5$.

Charges generated by QANBW are usually more energetic, but the probability of QANCS is higher, hence QANCS and QANBW are both important for QA.

## Simulations

A straightforward method to realize QA is to inject an electron bunch which can be generated by a laser plasma accelerator^2^ into a tightly focused UIL pulse. The electrons emit many *γ* photons, some of them annihilate into pairs and then generate more *γ* photons, etc. In such a cascade, QANCS and QANBW can generate high energy charges.

**Figure 6:**
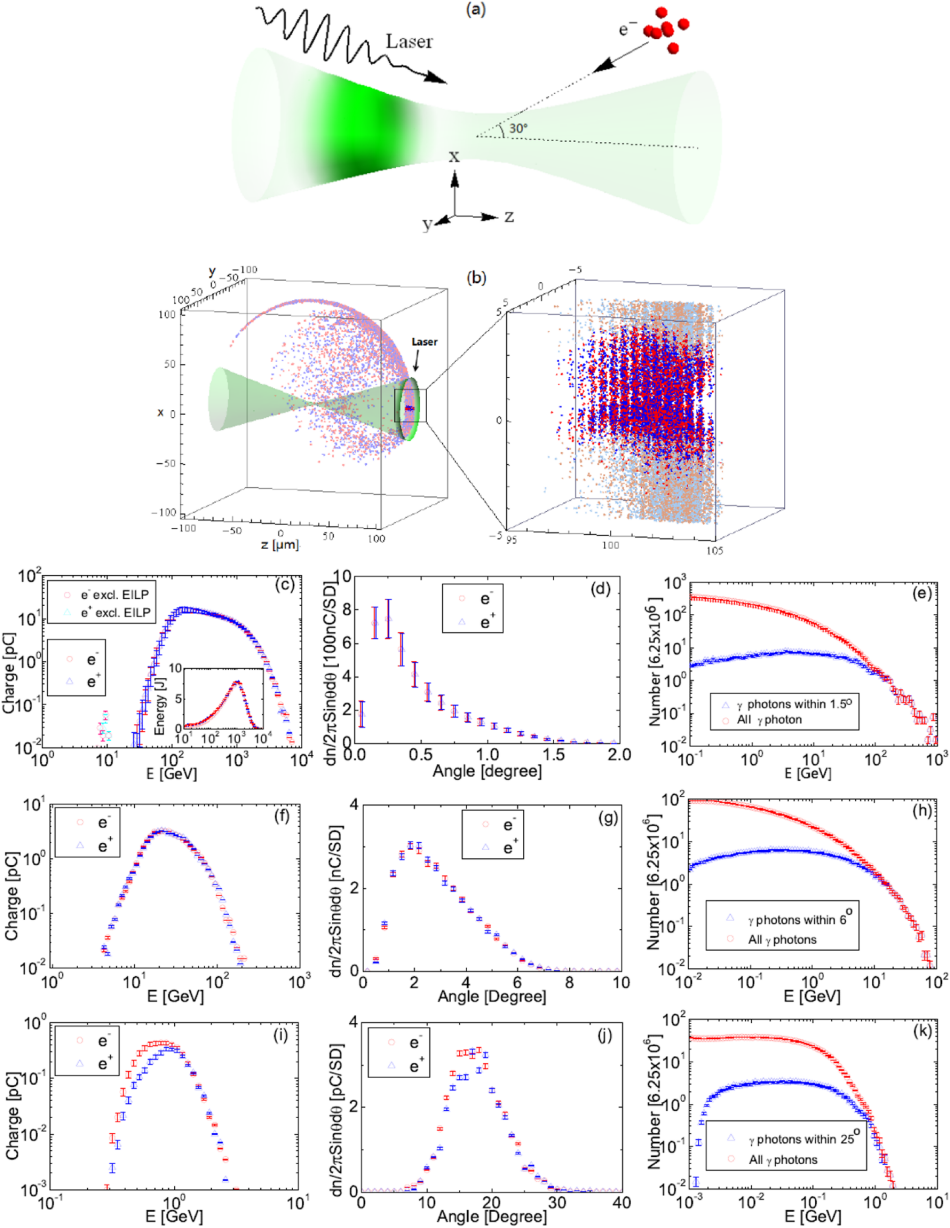
Schematics of QA simulation (**a**) and results (**b**–**k**). Spatial distribution of *e*^−^ s and *e*^+^*e*^+^ s (**b**) (*I*_0_ = 10^26^ W/cm^2^, *t* = 367 fs, bright red/blue for *e*^−^/*e*^+^ above 100 GeV and light ones for those below); Spectra of the generated *e*^−^-*e*^+^ beam (charges within ±5% energy range within 1.5° to the laser) (**c**); Angular distributions of *e*^−^ s and *e*^+^ s above 100 GeV (**d**); Spectrum of generated *γ* photons (**e**). Simulation results for proof-of-principle QA experiments were shown in (**f–h**) (*I*_0_ = 10^25^ W/cm^2^) and **(i**–**k**) (5 × 10^23^ W/cm^2^), the angle of the beams were 6° and 25°, and the angular distributions show that of *e*^−^ s and *e*^+^ s above 10 GeV and 1 GeV, respectively.

Monte-Carlo simulations were carried out to explore QA with such configuration. A schematics is shown in Fig. 6(a), the laser is linearly polarized along the *x* axis and the angle between the bunch and the laser is 30°.

NCS and NBW are included as instantaneous and local processes, the formulae including the effects of involved laser photons^13^ are included in *Method*. Between quantum processes, classical equations of motion describe Lorentz force and particle propagation. Other quantum processes and effects such as higher order radiations^17^, Schwinger pair production^18^, vacuum birefringence^19^, Unruh radiation^20^, vacuum radiation^21^, light bending^22^ and nonlinear trident process are negligible, see the Supplemental Information for details.

The ~100 kJ laser has a peak intensity of *I*_0_ = 1.37(*a*_0_/*λ*_0_[*μ*m])^2^ × 10^18^ W/cm^2^ = 10^26^ W/cm^2^, its wave length and radius are both 1 *μ*m and lasts *τ*_0_ = 2*λ*/*c* = 20 fs. The field of such tightly focused laser can be found in^23,26^. Electromagnetic force between the charges is ignored for it is $7 \sim 8$ orders weaker than the laser Lorentz force.

Most parameters of the electron bunch are adopted from ref. ^24^ its energy, charge, energy spread and angular dispersion are 2 GeV, 63 pC, 6% and 0.6 mrad. These electrons distribute uniformly in a *D* = 1 *μ*m sphere. The initial particle number is *N* = 10^5^, each represent 3937.5 electrons. The electron bunch and the laser pulse are both 10 *μ*m from the focus (located at the origin) at the beginning of the 367 fs (110 *μ*m/c) simulation and the time step is Δ*t* = 1.67 × 10^−2^ fs.

The simulation time allows most *e*^−^ and *e*^+^ below 100 GeV to escape the laser, *N* is large enough and Δ*t* is fine enough for the results to converge. Radiation is consistently considered since NCS itself is the major mechanism of radiation here, other radiation processes such as that caused by electron spin flip are negligible, details can be found in the Supplemental Information. Consumption of laser energy is negligible therefore induced laser field decrease is also ignored. The simulation errors, such as energy and direction variations in the co-propagation process after simulation are small. See the Supplemental Information for detailed estimations.

The interaction of the electron bunch with the pulse generates 1.156 nC *e*^−^ and 1.093 nC *e*^+^. The charges of *e*^−^/*e*^+^ above 100 GeV and 1 TeV are 353.7 pC/353.8 pC and 43.6 pC/43.5 pC. The interaction consumes 410 J of the laser energy, in which 350.3 J goes to charges above 100 GeV (140 J above 1 TeV), and 59.7 J is dissipated in *γ* photons and charges below 100 GeV. Considering only $\sim \mathrm{0.41 \% }$ of the laser energy was consumed, a much stronger beam can be generated through QA by injecting more electrons. The charge of the generated beam grows linearly with the seed as long as the laser is far from depletion.

Figure 6(b) shows the spatial distribution of *e*^−^ and *e*^+^ at the end of simulation (*t* = 367 fs), *e*^−^ s and *e*^+^ s are denoted as red and blue points (bright red/blue ones: >100 GeV, light red/blue ones: <100 GeV), respectively. The laser and its position at *t* = 367 fs are also shown to guide the eyes. All the charges above 100 GeV concentrate around the laser axis and are still co-propagating with the laser, forming a narrow quasi-neutral beam ($\sim {1.5}^{\circ }$). Figure 6(c) shows the spectra (charges within ±5% energy range) of *e*^+^ and *e*^−^ within 1.5°, it starts from $\lesssim 100$ GeV and ends at several TeV, and the energy distributions peaks around 1 TeV as shown in the inset. The major part of this spectra (>100 GeV) can be described by staged power-law. The angular distributions of *e*^−^ and *e*^+^ above 100 GeV shown in (d) also confirm the tight collimation of QA. A simple estimation give the angular dispersion of QA $\sim \,{a}_{0}m/{a}_{0}^{3}{k}_{0}$, which agrees well with (d). Note *e*^−^ and *e*^+^ are basically symmetric in the generated quasi-neutral beam (*ne*^+^/*n*_*e*−_ ≈ 0.95).

A simulation excluding the energy transferred from involved laser photons (EILP), i.e., adopt k′ ≈ *u*p/(1 + *u*), p′ ≈ p/(1 + *u*), p′′ ≈ *δ*k′′ and p′′′ ≈ (1−*δ*)k′′^11,12^ which are good approximations at lower intensities when $n{k}_{0}\ll {p}_{0}$ and $n{k}_{0}\ll {k}_{0}^{^{\prime\prime} }$, were also performed for comparison. Generated *e*^−^ and *e*^+^ are below 20 GeV and only a few of them are within the 1.5° cone, corresponding results are shown in Fig. 6(c). It proves that the TeV scale beam is generated by QA rather than Lorentz force.

Simulations also show that proof of principle QA experiment is possible on 100 PW scale lasers. The geometry of the simulation is similar, and the electron bunch parameters are adopted from ref. ^25^ the energy, charge, angular dispersion and energy spread are 1 GeV, 30 pC, 1.6 mrad and 2.5%. The peak intensity of the laser *I*_0_ = 5 × 10^23^ W/cm^2^ is possible on SEL if the laser is focused to 3−5 *μ*m^10^. The simulation time is extended to 10033 fs to reduce simulation errors to acceptable level. QA generates an *e*^−^ − *e*^+^ beam up to 3 GeV within $\sim {25}^{\circ }$ to the laser. The spectrum of this beam (<25°) and the angular distribution of charges above 1 GeV are shown in Fig. 6(i,j). By contrast, if the energy of involved laser photons are excluded, there is no charge within 25° and the spectra of *e*^−^ and *e*^+^ end at 0.8 GeV. An additional simulation shows that if the 100 PW laser can be ideally focused to 1 *μ*m to reach 10^25^ W/cm^2^, QA can generate an *e*^−^ − *e*^+^ beam up to 200 GeV within 6° to the laser. The spectrum of the beam within 6° and the angular distribution of all charges above 10 GeV are shown in Fig. 6(f,g).

The beam-UIL interaction process is like an avalanche, it needs both QANCS and QANBW. QANCS generates *γ* photons and has a comparatively small chance to boost the charges to high energy. QANBW turn the *γ* photon into charges and has higher probability to generate high energy charges. Comparatively low energy charges generated by QANBW experience more QANCS, etc. Hence both QANCS and QANBW are important for QA.

## Conclusions

Two quantum acceleration methods, QANCS and QANBW, i.e., QA through NCS and NBW in coherent photon dominated regime were proposed. QANCS transfers and QANBW converts the energy of coherent laser photons to high energy *e*^−^ s and *e*^+^ s effectively through quantum processes and collimate them along the laser axis, but heavier charges are not suitable for QA in foreseeable future. Charge procreation generates a *e*^−^-*e*^+^ quasi-neutral beam and greatly enhances the beam flux.

QA has a microscopic effective acceleration distance, all the high energy charges are generated within the Rayleigh length of UIL, which is usually of *μ*m scale. This is many orders shorter than conventional accelerators (km scale) or staged 100 GeV laser-plasma accelerators in plan (100 m scale)^29^.

The energy scale of QA $\sim \,{a}_{0}^{3}{k}_{0}\propto {I}_{0}^{\mathrm{3/2}}{\lambda }_{0}^{2}$ and the angular dispersion $\sim {a}_{0}m/{a}_{0}^{3}{k}_{0}\propto {I}_{0}^{-1}{\lambda }_{0}^{-1}$ rely nonlinearly on both laser intensity and wavelength. Lasers with longer wavelengths or higher intensities can generate beams with higher energy and smaller angular dispersion through QA.

Classical accelerators are of series structures, charges fly through linear arrays of blocks and are propelled by repeated fields inside. In contrast, quantum accelerators are of parallel structures. In foreseeable future, UILs of 100 PW scale and above would be realized by combining coherent laser pulses generated by parallel laser blocks, e.g., SEL = 4 × 25 PW. In general, one can combine more parallel beams to realize higher energy QA.

Laser wake field acceleration (LWFA) is an intensively studied new method of particle acceleration based on laser and plasma. Compared with QA which accelerates positrons naturally, positron acceleration is still a very challenging task for LWFA so far. To reach the energy frontiers of high energy physics (100 GeV to TeV), LWFA need multiple stages, but efficient coupling between adjacent stages is still very hard. In contrast, QA has no stage coupling problem.

Simulations showed that EW scale facilities are natural quantum accelerators and proof of principle QA experiment can be performed on 100 PW scale lasers. QA is an effect of involved laser photons, and the latter can be tested on 10 PW scale lasers^13^. The alignment of electron bunches and laser pulses is challenging. Presently, experiments of other purposes can achieve in $\sim \mathrm{50 \% }$ of the shots^30^. The monochromaticity of QA also need to be improved in the future.

Finally, direct laser acceleration (DLA) appears possible to reach the energy scale of *a*_0_^2^*m* using UILs by keeping it focused and propagate in a tube^27^. However, when laser intensity reaches 10^24^ W/cm^2^ or higher, radiation damping and depletion of laser^15,28^ are too strong in plasmas.

## Method

The major mechanism of electron/positron emission in UILs (${a}_{0}\gg 1$) is NCS. When ${a}_{0}\gg 1$, the coherence interval of NCS is very short ($\delta \phi  \sim \mathrm{1/}{a}_{0}$), hence NCS can be considered as local and instantaneous processes in UILs. Its emission rate is

8 $$\begin{array}{rcl}\frac{d{N}_{NCS}}{dudt} & = & \frac{dW}{dudt}={F}_{\chi ,{p}_{0}}(u)\\  & = & \frac{\alpha }{\pi \sqrt{3}}\frac{{m}^{2}}{{p}_{0}}\frac{1}{{(1+u)}^{2}}[(1+u+\frac{1}{1+u}){K}_{2/3}(\frac{2u}{3\chi })\\  &  & \,-{\int }_{2u/3\chi }^{\infty }\,dy{K}_{1/3}(y)],\end{array}$$

where $\chi =e\sqrt{-{({F}_{\mu \nu }{p}^{\nu })}^{2}}/{m}^{3}$, *K*_*ν*_(*x*) is the modified Bessel function of the *ν* th order, *α* is the fine structure constant, *p*_0_ is the electron energy and *u* = *kk*′/*kp*′.

When $n{k}_{0}\ll {p}_{0}$, the energy (and momentum) of involved laser photons can be ignored, k′ ≈ *u*p/(1 + *u*) and p′ ≈ p/(1 + *u*). In the opposite case ($n{k}_{0}\gtrsim {p}_{0}$) which is important for QANCS, the effects of involved laser photons are very important^13^. In the frame that *p* on the *z* axis and *k* on the *x* − *z* plane,

9 $$\begin{array}{rcl}{\rm{p}}{\text{'}}_{\pm } & \approx  & \frac{\mathrm{(1}+{u}^{2}C){p}_{0}}{1+u}\\  &  & \,(\begin{array}{c}\sin \,\theta {\rm{s}}{\rm{i}}{{\rm{n}}}^{2}\frac{{\theta }_{p^{\prime} }^{B}}{2}\pm \,\sin \,\frac{\theta }{2}\,\cos \,\phi \,\sin \,{\theta }_{p^{\prime} }^{B}\\ \mp \,\sin \,\frac{\theta }{2}\,\sin \,{\theta }_{p^{\prime} }^{B}\,\sin \,\phi \\ 1-2\,{\sin }^{2}\frac{{\theta }_{p^{\prime} }^{B}}{2}{\sin }^{2}\frac{\theta }{2}\pm \,\cos \,\phi \,\cos \,\frac{\theta }{2}\,\sin \,{\theta }_{p^{\prime} }^{B}\end{array})\mathrm{}.\end{array}$$

and

10 $$\begin{array}{rcl}{\rm{k}}{\text{'}}_{\pm } & \approx  & \frac{u\mathrm{(1}+C){p}_{0}}{1+u}\\  &  & \,(\begin{array}{c}\sin \,\theta \,{\sin }^{2}\frac{{\theta }_{k^{\prime} }^{B}}{2}\mp \,\sin \,\frac{\theta }{2}\,\cos \,\phi \,\sin \,{\theta }_{k^{\prime} }^{B}\\ \pm {\rm{s}}{\rm{i}}{\rm{n}}\frac{\theta }{2}\,\sin \,{\theta }_{k^{\prime} }^{B}\,\sin \,\phi \\ 1-2\,{\sin }^{2}\frac{{\theta }_{k^{\prime} }^{B}}{2}{\rm{s}}{\rm{i}}{{\rm{n}}}^{2}\frac{\theta }{2}\mp \,\cos \,\phi \,\cos \,\frac{\theta }{2}\,\sin \,{\theta }_{k^{\prime} }^{B}\end{array})\mathrm{}.\end{array}$$

where *C* = *a*_0_^3^*k*_0_/*χp*_0_,

11 $$\begin{array}{rcl}\cos \,{\theta }_{p^{\prime} }^{B} & \approx  & \frac{1-{u}^{2}C}{1+{u}^{2}C}\\ \cos \,{\theta }_{k^{\prime} }^{B} & \approx  & \frac{1-C}{1+C},\end{array}$$

*θ* is the angle between k and p and ϕ is the angle from *x* − *z* plane to the polarization vector a. The ± subscripts are the sign of

12 $$\rho =\frac{e{F}^{\mu \nu }{p}_{\mu }^{^{\prime} }{p}_{\nu }}{{m}^{2}{a}_{0}^{2}kk^{\prime} },$$

The chances for both signs are 1/2 because the differential probability is a even function of *ρ*. Details can be found in ref. ^14^.

The pair production rate

13 $$\begin{array}{c}\frac{d{N}_{NBW}}{dtd\delta }=\frac{d{W}_{\gamma }}{dtd\delta }\approx \frac{\alpha {m}^{2}}{\sqrt{3}\pi {k}_{0}^{^{\prime} }}[(\frac{1-\delta }{\delta }+\frac{\delta }{1-\delta }){K}_{\mathrm{2/3}}(\kappa )\\ \,-{\int }_{\kappa }^{\infty }\,{K}_{\mathrm{1/3}}(y)dy]\mathrm{}.\end{array}$$

where $$\chi ^{\prime} =e\sqrt{-{({F}_{\mu \nu }k{^{\prime\prime} }^{\nu })}^{2}}/{m}^{3}$$, $$\kappa =\frac{2}{3\chi ^{\prime} \delta (1-\delta )}$$ and $$\delta =\frac{kp^{\prime\prime} }{kk^{\prime\prime} }$$.

Similarly, when $n^{\prime} {k}_{0}\ll {k^{\prime\prime} }_{0}$, effects of involved laser photons is not important, *p*′′ ≈ *δk*′′ and *p*′′′ ≈ (1−*δ*)*k*′′. The opposite case ($n^{\prime} {k}_{0}\gtrsim {k^{\prime\prime} }_{0}$) is important for QANBW^14^, in this case, the emitted electron momentum is

14 $$\begin{array}{rcl}{{\rm{p}}^{\prime\prime} }_{\pm } & = & \frac{({k^{\prime\prime} }_{0}+n^{\prime} {k}_{0})}{2}(1+(2\delta -1)\zeta )\\  &  & \,(\begin{array}{c}\sin \,\theta \,{\sin }^{2}\frac{{\theta }_{p^{\prime\prime} }^{B}}{2}\mp \,\sin \,\frac{\theta }{2}cos\,\phi \,\sin \,{\theta }_{p^{\prime\prime} }^{B}\\ \pm \,\sin \,\frac{\theta }{2}\,\sin \,{\theta }_{p^{\prime\prime} }^{B}{\rm{s}}{\rm{in}}\,\phi \\ 1-2\,{\sin }^{2}\frac{{\theta }_{p^{\prime\prime} }^{B}}{2}{\sin }^{2}\frac{\theta }{2}\mp \,\cos \,\frac{\theta }{2}\,\cos \,\phi \,\sin \,{\theta }_{p^{\prime\prime} }^{B}\end{array}),\end{array}$$

and that of *e*^+^ is

15 $$\begin{array}{c}\begin{array}{rcl}{{\rm{p}}^{\prime\prime} }_{\pm } & = & \frac{({k^{\prime\prime} }_{0}+n^{\prime} {k}_{0})}{2}(1-(2\delta -1)\zeta )\\  &  & \,(\begin{array}{c}\sin \,\theta \,{\sin }^{2}\frac{{\theta }_{p^{\prime\prime\prime} }^{B}}{2}\pm \,\sin \,\frac{\theta }{2}\,\cos \,\phi \,\sin \,{\theta }_{p^{\prime\prime\prime} }^{B}\\ \mp \,\sin \,\frac{\theta }{2}\,\sin \,{\theta }_{p^{\prime\prime\prime} }^{B}\,\sin \,\phi \\ 1-2\,{\sin }^{2}\frac{\theta }{2}{\sin }^{2}\frac{{\theta }_{p^{\prime\prime\prime} }^{B}}{2}\pm \,\cos \,\phi \,\cos \,\frac{\theta }{2}\,\sin \,{\theta }_{p^{\prime\prime\prime} }^{B}\end{array}),\end{array}\end{array}$$

in the frame that k″ is on the *z* axis and k on the *x* − *z* plane, where

16 $$\begin{array}{rcl}\cos \,{\theta }_{{p}^{^{\prime\prime} }}^{B} & = & \frac{\zeta +\mathrm{(2}\delta -\mathrm{1)}}{1+\mathrm{(2}\delta -\mathrm{1)}\zeta }\\ \cos \,{\theta }_{{p}^{^{\prime\prime\prime} }}^{B} & = & \frac{\zeta -\mathrm{(2}\delta -\mathrm{1)}}{1-\mathrm{(2}\delta -\mathrm{1)}\zeta },\end{array}$$

and

17 $$\zeta =\frac{{k}_{0}^{^{\prime\prime} }-n^{\prime} {k}_{0}}{{k}_{0}^{^{\prime\prime} }+n^{\prime} {k}_{0}},$$

*θ* is the angle between k and k′′ and ϕ is the angle from *x* − *z* plane to the polarization vector *a*.

Again, ± subscripts are the sign of

18 $$\rho ^{\prime} =\frac{e{F}^{\mu \nu }{p^{\prime\prime\prime} }_{\mu }{p^{\prime\prime} }_{\nu }}{{m}^{2}{a}_{0}^{2}kk^{\prime\prime} }$$

and both signs take half of the chance. Details can be found in ref. ^13^.

The simulation considers Lorentz force, NCS and NBW. In every time step, NCS happens to an electron/positron when *dW*_*NCS*_/*dt*Δ*t* ≥ *n*_*r*_, where *n*_*r*_∈[0, 1] is a uniformly distributed random number. NBW is included in a similar way.

The scattering between an electron and incoherent photons is (linear) Compton scattering:

19 $${e}^{-}(p)+\gamma (k)\to {e}^{-}({p^{\prime} }_{L})+\gamma ({k^{\prime} }_{L}),$$

where *p*, *k*, *p*′_*L*_ and *k*′_*L*_ are corresponding 4-momentum, subscript L stands for linear Compton scattering. Averaged over the spins, the amplitude of Compton scattering has^16^

20 $$\begin{array}{c}\frac{1}{4}\sum _{spins}| {\mathcal M} {|}^{2}=2{e}^{4}[\frac{pk^{\prime} }{pk}+\frac{pk}{pk^{\prime} }+2{m}^{2}(\frac{1}{pk}-\frac{1}{pk^{\prime} })\\ \,+{m}^{4}{(\frac{1}{pk}-\frac{1}{pk^{\prime} })}^{2}].\end{array}$$

The total cross section has simple expressions in two limits. When *s* = (*p* + *k*)^2^ approaches its minimum of *m*^2^, the cross section

21 $${\sigma }_{total}{|}_{s\to {m}^{2}}\approx \frac{8\pi {\alpha }^{2}}{3{m}^{2}}.$$

In the opposite case, when *s* = (*p* + *k*)^2^ ≫ *m*^2^,

22 $${\sigma }_{total}{|}_{s\gg {m}^{2}}\approx \frac{2\pi {\alpha }^{2}}{s}\,\mathrm{ln}\,(\frac{s}{{m}^{2}}).$$

## Supplementary information

Supplementary Information

## Data Availability

The data that support the findings of this study are available from the corresponding authors on reasonable request.
