# Supplementary material for: Quantum Mechanisms of Electron and Positron Acceleration through Nonlinear Compton Scatterings and Nonlinear Breit-Wheeler Processes in Coherent Photon Dominated Regime
Source: Sci Rep. 2019 Dec 11;9:18876. doi: 10.1038/s41598-019-55472-5 (PMC6906303; doi:10.1038/s41598-019-55472-5)
Supplement: Supplementary file 1 — Supplementary Information [file 41598_2019_55472_MOESM1_ESM.pdf]

Supplemental Information for "Quantum Mechanisms of Electron and Positron Acceleration through Nonlinear Compton Scatterings and Nonlinear Breit-Wheeler Processes in Coherent Photon Dominated Regime "

Bo Zhang,[1,2,*] Zhimeng Zhang,[1,2] Zhi-gang Deng,[1] Jian Teng,[1]
Shu-kai He,[1] Wei Hong,[1,2] Weimin Zhou,[1,2] and Yuqiu Gu[1,2,†]

[1] *Department of High Energy Density Physics, Research Center of Laser Fusion, 621900, Mianyang, Sichuan, China*
[2] *Laboratory of Science and Technology on Plasma Physics, Research Center of Laser Fusion, 621900, Mianyang, Sichuan, China*

(Dated: November 5, 2019)

I. OTHER QUANTUM PROCESSES AND THEIR EFFECTS

In the simulation, only two SFQED processes were considered, nonlinear Compton scattering (NCS) and nonlinear Breit-Wheeler process (NBW). Effects of other SFQED processes such as higher order radiations, Schwinger pair production, vacuum birefringence, Unruh radiation, vacuum radiation, light bending and nonlinear trident process are negligible in the laser-electron beam interaction considered. We will analyze them one by one below.

Higher order NCS and NBW are similar to NCS and NBW but release more than one photon/pair. The ratio between the probability of emitting n photons in one coherence interval and that of releasing one photon is of the order of $(\alpha\chi^{2/3})^{n-1}$ when $\chi \gg 1$ [1] and is of the order of α^{n-1} when $\chi \lesssim 1$. The condition for double NCS and even higher order processes to be ignorable is then $\chi \ll 10^3$, and the condition for higher order NBW is similar. In theory, the χ has an upper limit of $\chi_0 = 2p_0 a_0^{peak} k_0/m^2 \approx 290$ in the simulation (the $I = 10^{26}\mathrm{W/cm^2}$ case). Apparently, it satisfies the condition of $\chi \ll 10^3$. Furthermore, due to the emissions and pair productions before the particles reach the laser peak, the χ and χ_γ only reached ~ 30 in the simulation. Other higher order nonlinear quantum processes such as nonlinear trident process shown in Fig. 1 are suppressed by a factor of $(\alpha\chi^{2/3})^{n-1}$ for the same reason.

Vacuum radiation is a newly found SFQED process, a time dependent electric field gives the virtual electron-positron fluctuation in the vacuum a non-vanishing energy. This energy is then released by radiating a pair of entangled photon. According to the formulae in Ref. [2], the energy of emitted photons under the field conditions we consider is ~ 0.1 eV and the expectation of produced photon number is less than 1. Vacuum radiation is hence ignorable in our simulations.

Vacuum birefringence modifies Maxwell's equations and influences the propagation of electromagnetic waves

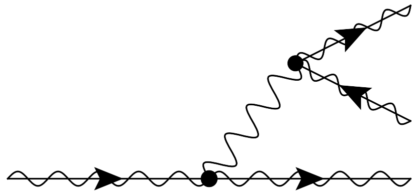

FIG. 1. Diagram for nonlinear trident process.

[3]. The modified Maxwell's equations in the vacuum are

$$\begin{cases} \nabla\cdot\mathbf{E} = -\nabla\cdot\mathbf{P} \\ \nabla\times\mathbf{B} - \dfrac{\partial\mathbf{E}}{dt} = \nabla\times\mathbf{M} + \dfrac{\partial\mathbf{P}}{dt} \\ \nabla\cdot\mathbf{B} = 0 \\ \nabla\times\mathbf{E} + \dfrac{\partial\mathbf{B}}{dt} = 0 \end{cases}, \quad (1)$$

where the field induced vacuum polarization $\mathbf{P}$ and vacuum magnetization $\mathbf{M}$ are

$$\mathbf{P} = \frac{4\alpha^2}{45m^4}[2(E^2 - B^2)\mathbf{E} + 7(E\cdot B)\mathbf{B}]$$
$$\mathbf{M} = \frac{4\alpha^2}{45m^4}[2(E^2 - B^2)\mathbf{B} - 7(E\cdot B)\mathbf{E}] \quad (2)$$

to the first order of vacuum birefringence effects. The additional terms in the modified Maxwell's equations are proportional to $F^{\mu\nu}F_{\mu\nu} \propto E^2 - B^2$ or $\tilde{F}^{\mu\nu}F_{\mu\nu} \propto E\cdot B$ and both terms vanish in plane waves laser. In a tightly focused laser, these two terms do not completely vanish but are much small than E^2. According to the formulae for tightly focused laser fields [6] which are also included in Sec. III of this supplemental information, the maximum of $(E^2 - B^2)/E^2$ and $E\cdot B/E^2$ are ~ 0.01 in the laser we consider. Hence vacuum birefringence induced modification to the phase of laser is within 10^{-8} and refractions of both the laser and the γ photons are within 10^{-7} rad. As a result, vacuum birefringence effects are ignorable in our simulations.

Virtual electron-positron fluctuation can obtain enough energy in extremely strong electric fields ($E \sim m^2/e$) and produce real electron-positron pairs, this process is usually called Schwinger pair production (SPP)

* zhangbolfrc@caep.cn
† yqgu@caep.cn

[4]. The probability of SPP

$$\frac{dW_{SPP}}{dx^3dt} = \frac{\alpha^2}{\pi^2}\mathcal{E}^2 e^{-\frac{\pi m^2}{e\mathcal{E}}} \quad (3)$$

where m and e are electron mass and charge, α is the fine structure constant and $\mathcal{E} = \sqrt{E^2 - B^2}$. Apparently, SPP rate is strongly suppressed by the laser magnetic field. Put the field of tightly focused laser in section III in Eq. (3), the expectation of SPP in the laser field considered in our simulation is below 10^{-400}/shot, which is apparently ignorable.

Unruh radiation is the emission of an entangled photon pair by an accelerating charge [5]. The temperature of Unruh radiation is

$$T = \frac{a}{2\pi} \quad (4)$$

where a is the acceleration. For laser-electron interaction, the upper limit of acceleration $a_{max} = e(E + v \times B)/\gamma m$ is $a_0^{max}k_0$, this maximum is reached when an electron is almost stationary at the peak of the laser. In our simulation, the corresponding upper limit of Unruh radiation temperature is $\sim$ 1keV. Since the energies of most electrons are above 1GeV in the simulation, the typical Unruh radiation temperature in the interactions we considered with simulations is below 1eV. Hence Unruh radiation can be neglected in our simulations.

NCS is the major radiation process in UILs, but there are other radiation processes, e. g., electron spin flip. Since the strongest magnetic field we considered is below $0.02E_S/c$, the energy difference between electron spin states is of 10keV scale. Compared with NCS radiations from 100MeV to 1TeV scale in our simulation, radiations due to spin flips are negligible.

II. IMPERFECT VACUUM

Vacua in target chambers of laser laboratories is usually created with molecular pumps, usually, the pressure in target chambers of UILs can reach 10^{-3}Pa. At such pressure, the molecule density is $\sim 0.27/\mu$m^3 and the electron density is $\sim 2/\mu$m^3. Hence the number of electrons ionized from molecules by laser at the focus spot is much less than that of the injected beam. The influences of these electrons with ionization origin are negligible. Furthermore, ionized electrons will be QAed like the injected ones.

III. FIELDS

In our simulation, we adopt the formulae for tightly focused laser field in Ref. [6], which were expanded in details in the supplemental material of Ref. [7]. The electric field is

$$\begin{aligned}
E_x &= \frac{-E_1}{C_1^2}\big[C_1^3 - f^2C_1\frac{x^2}{z_r^2} + \frac{fC_1}{k_0z_r} - \frac{2ifk_0x^2}{z_r(2k_0j_z+\eta)^2}\big]\\
E_y &= \frac{E_1fxy}{C_1^2z_r^2}\big[fC_1 + \frac{2ik_0z_r}{(2k_0j_z+\eta)^2}\big]\\
E_z &= \frac{-E_1fx}{C_1^2z_r^2}\big[\frac{ifC_1}{k_0} - C_1z_r(i + \frac{if^2r^2}{4z_r^2} - \frac{\eta}{s^2})\\
&\quad -\frac{iC_1z_r}{s^2+i\eta} - z_rC_2\big],
\end{aligned} \quad (5)$$

and the magnetic field is

$$\begin{aligned}
B_x &= 0\\
B_y &= E_1\big(\frac{if}{2k_0z_r} - i - \frac{if^2r^2}{4z_r^2} + \frac{\eta}{s^2} + \frac{1}{is^2-\eta}\big)\\
B_z &= \frac{E_1fy}{z_r}.
\end{aligned} \quad (6)$$

The parameters are

$$\begin{aligned}
E_1 &= \frac{E_0\psi e^{i\eta}}{S_0}\\
S_0 &= \frac{-s_1^2 - \frac{1}{k_0z_r}}{s_1}\\
s_1 &= i[1 + 1/(2k_0z_r) + 1/s^2]\\
j_z &= z + iz_r\\
C_1 &= i + \frac{ik_0^2r^2 - 2k_0j_z - \eta}{(2k_0j_z+\eta)^2} + \frac{s^2 + i\eta s^2 - \eta^2}{s^2(\eta - is^2)}\\
C_2 &= \frac{-1}{(2k_0j_z+\eta)^2} + \frac{4k_0j_z + 2\eta - 2ik_0^2r^2}{(2k_0j_z+\eta)^3}\\
&\quad + \frac{\eta^2 - s^2 - i\eta s^2}{(i\eta s + s^3)^2} + \frac{2i\eta + s^2}{i\eta s^2 + s^4}\\
\psi &= f(1 + \frac{i\eta}{s^2})\exp[i\phi_0 - f\rho^2 - \frac{\eta^2}{2s^2}]\\
f &= \frac{i}{i + \nu/z_r}\\
\nu &= \frac{z+t}{2}\\
\eta &= k_0(t-z)\\
\rho &= r/w_0\\
r &= \sqrt{x^2+y^2}\\
s &= \omega_0\tau_0/2\sqrt{2}\\
z_r &= k_0w_0^2/2
\end{aligned} \quad (7)$$

where E_0, w_0, k_0 and τ_0 are maximal electric field, laser beam waist, frequency and duration of the laser.

IV. SIMULATION ERRORS

At the end of our simulations, the laser pulse and the co-propagating high energy electron-positron beam are

about 100μm from the focus ($I_{peak} = 10^{26}$W/cm^2 simulations), or about 3000μm from the focus (simulations with $I_{peak} = 10^{25}$W/cm^2 or 5×10^{23}W/cm^2).

Since the high energy electrons and positrons are collimated by QA along the laser axis, they still interact with the laser after the simulations. Then what are the simulation errors, i. e., differences between the beam at the end of simulation and the beam separated from the laser by a mirror placed at an appropriate distance? As will be proved in this section, the simulation errors for the generated beam along the laser axis are small enough.

A. Lorentz force

First, we estimate the influence of Lorentz force. For a high energy electron co-propagating with the laser pulse and with a very small angle θ to the axis, the longitudinal component of the Lorentz force is approximately θeE and the transverse component is approximately $\theta^2 eE/2$.

At a point far away from the laser focus ($l \gg z_r$, the Rayleigh length) near the laser axis, the maximal field strength

$$E_{max}(l) \approx E_0 z_r / l \tag{8}$$

where E_0 is the maximal laser electric field at the focus and l is the longitudinal distance to the focus.

The energy change ΔE in the co-propagation process of an electron/positron from the end of simulation to the mirror has an upper limit of

$$eE_0 z_r \theta \int_{l_0}^{\min[l_r,(1-\phi/\pi)\lambda/\theta^2+l_0]} \frac{dl}{l} \sin(kl\theta^2/2 + \phi), \tag{9}$$

where ϕ is the initial phase with respect to the nearest node behind the electron/positron, l_0 is the distance to the focus at the end of simulation and l_r the distance to the mirror.

Similarly, the upper limit for transverse momentum change $\Delta p_\perp$ is

$$\frac{eE_0 z_r \theta^2}{2c} \int_{l_0}^{\min[l_r,(1-\phi/\pi)\lambda/\theta^2+l_0]} \frac{dl}{l} \sin(kl\theta^2/2 + \phi) \tag{10}$$

Under the laser conditions we considered, set $l_r = 1$m, the upper limit for electron/positron energy and transverse momentum change in the $I_{peak} = 10^{26}$W/cm^2 simulation with $l_0 = 100\mu$m is within 4.5GeV and 0.06GeV/c, respectively. Hence the energy and direction errors for an electron/positron in the beam (> 100GeV) are within 5% and 0.1%. In the simulations with $I_{peak} = 10^{25}$W/cm^2 and $I_{peak} = 5 \times 10^{23}$W/cm^2, the simulation lasts $t_s = l_0/c = 3000\mu$m/c, and corresponding upper limits are 0.13GeV, 1.5MeV/c and 0.06GeV, 0.7MeV/c, all negligible in corresponding simulations. Note that the errors for co-propagating positrons are the same and these errors are for a single electron/positron.

Considering that some electrons/positrons in the beam obtain energies in the co-propagation process while some loss energies, the errors for the beam should be much smaller than that for a single electron/positron. For similar reasons, the variation of beam divergence angle is also much smaller than that for a single electron/positron.

B. radiation

On the way from l_0 to l_r, the main mechanism for an electron to radiate is NCS. Then it is important to know how many times an electron/positron radiates in the co-propagation process and the effect of the radiations on the electron/positron.

The expectation of radiations from l_0 to l_r is

$$\int_{l_0}^{l_r} \frac{dl}{c} \frac{dW}{dt}(\chi, p_0). \tag{11}$$

Include the phase ϕ in the last subsection and the effect of pulse duration τ_0, one can obtain that the maximal value of the expectation of radiations is 2.1 in the $I_{peak} = 10^{26}$W/cm^2 simulation.

Then what is the influence of radiations on the electron? After the simulation, the involved energy of laser photons in one scattering is greatly reduced and $nk_0 \ll p_0$ is satisfied in the co-propagation process. The model for NCS in Ref. [8, 9] is then suitable to describe radiations in the co-propagation, i. e., the radiation is forward and the electron recoils. The energy an electron losses in a radiation approximately $0.44\chi p_0$ [10]. Then the energy loss rate due to radiation during the co-propagation process from l_0 to l_r is

$$\int_{l_0}^{l_r} \frac{dl}{c} \frac{dW}{dt}(\chi, p_0) 0.44\chi. \tag{12}$$

Include the influence of ϕ and τ_0, the maximal radiation loss rate is < 1% in the $I_{peak} = 10^{26}$W/cm^2 simulation. Hence radiation loss in the co-propagation process is negligible. In simulations with $I_{peak} = 10^{25}$W/cm^2 or 5×10^{23}W/cm^2, the maximal radiation loss rates in the co-propagation process are further suppressed by several orders, mainly due to much longer simulation time and smaller χs.

Besides radiation in the co-propagation process, radiation loss is also possible at the reflection point at $l_r = 1$m. For electrons/positrons in the beam, when penetrate the reflected pulse, the expectation of radiation number is 2.7×10^{-3} and the radiation loss rate is < 0.01%.

Radiations in the co-propagation process and near the mirror both point forward, the reaction on the electron is approximately a recoil hence do not modify the direction of the electron/positron.

C. simulation time step

To prove the convergence of our simulations, we re-simulated the QA processes with shorter time steps (Δt is reduced from $T/200$ to $T/800$), where T is the laser period. These simulations with shorter time step confirmed the convergence of our simulations.

V. THE COLLIMATION INDUCED BY COHERENT PHOTON BOOST

The collimation induced by CPB can be explained quantitatively. In a CPB, $\chi' = \chi/1{+}u$ and $\chi_\gamma = u\chi/1{+}u$ satisfy $\chi' + \chi_\gamma = \chi$. The CPB collimated electron angle θ' then satisfies

$$(1 - \cos\theta') = \frac{m^2\chi}{p_0' a_0 k_0 (1+u)}, \tag{13}$$

Hence it decreases monotonically with $\hat{W}$. When $\hat{W}$ is close to 1, the electron obtains a high energy through CPB (large p_0'), therefore the collimation angle is very small. This is exactly the case observed and shown in Fig. 4 (a) and (b).

[1] Di Piazza, A., Hatsagortsyan, K. Z., & Keitel C. H., Quantum Radiation Reaction Effects in Multiphoton Compton Scattering. *Phys. Rev. Lett.* **105**, 220403 (2010).

[2] Zhang, B., Zhang, Z. M., Hong, W., Teng, J., He, S. K. & Gu, Y. Q. *Phys. Lett. B* **767**, 431-436 (2017).

[3] Klein, J. J. & Nigam, B. P. *Phys. Rev.* **135**, 1279-1280 (1964).

[4] Schwinger, J. *On gauge invariance and vacuuwn polarization. Phys. Rev.* **82**, 664-679 (1951).

[5] Unruh, W. G. Notes on black-hole evaporation. *Phys. Rev. D* **14**, 870-892 (1976).

[6] Esarey, E., Sprangle, P., Pilloff, M. & Krall, J. Theory and group velocity of ultrashort, tightly focused laser pulses. *J. Opt. Soc. Am. B* **12**, 1695 (1995).

[7] Li, J. X., Hatsagortsyan K. Z. & Keitel, C. H. Robust signatures of quantum radiation reaction in focused ultrashort laser pulses. *Phys. Rev. Lett.* **113**, 044801 (2014).

[8] Arber, T. D., et al. Contemporary particle-in-cell approach to laser-plasma modelling. *Plasma Phys. Control Fusion* **57**, 113001 (2015).

[9] Ridgers, C. P. Modelling gamma-ray photon emission and pair production in high-intensity lasermatter interactions. *J. Comp. Phys.* **260**, 273-285 (2014).

[10] Bell, A. R. & Kirk, J. G. Possibility of Prolific Pair Production with High-Power Lasers. *Phys. Rev. Lett.* **101**, 200403 (2008).
